# Involvement of MAPKs and PLC Pathways in Modulation of Pacemaking Activity by So-Cheong-Ryong-Tang in Interstitial Cells of Cajal from Murine Small Intestine

**DOI:** 10.1155/2013/536350

**Published:** 2013-10-30

**Authors:** Min Woo Hwang, Hee Jung Lee, Ho Joon Song, Byung Joo Kim

**Affiliations:** ^1^Department of Sasang Constitutional Medicine, College of Korean Medicine, Kyung Hee University, Seoul 130-701, Republic of Korea; ^2^Division of Longevity and Biofunctional Medicine, Pusan National University School of Korean Medicine, Beomeori, Mulgeum-eup, Yangsan, Gyeongsangnam-do 626-870, Republic of Korea

## Abstract

*Purpose*. Interstitial cells of Cajal (ICCs) are the pacemaker cells that generate slow waves in the gastrointestinal (GI) tract. We have aimed to investigate the effects of Socheongryong-Tang (SCRT) in ICCs from mouse's small intestine. *Methods*. The whole-cell patch-clamp configuration was used to record membrane potentials from cultured ICCs. Intracellular Ca^2+^ ([Ca^2+^]_i_) increase was studied in cultured ICCs using fura-2 AM. *Results*. ICCs generated pacemaker potentials in mouse's small intestine. SCRT produced membrane depolarization in current clamp mode. Y25130 (5-HT_3_ receptor antagonist) and RS39604 (5-HT_4_ receptor antagonist) blocked SCRT-induced membrane depolarizations, whereas SB269970 (5-HT_7_ receptor antagonist) did not. When GDP-**β**-S (1 mM) was in the pipette solution, SCRT did not induce the membrane depolarizations. [Ca^2+^]_i_ analysis showed that SCRT increased [Ca^2+^]_i_. In the presence of PD98059 (p42/44 MAPK inhibitor), SCRT did not produce membrane depolarizations. In addition, SB203580 (p38 MAPK inhibitor) and JNK inhibitors blocked the depolarizations by SCRT in pacemaker potentials. Furthermore, the membrane depolarizations by SCRT were not inhibited by U-73122, an active phospholipase C (PLC) inhibitor, but by U-73343, an inactive PLC inhibitor. *Conclusion*. These results suggest that SCRT might affect GI motility by the modulation of pacemaker activity through MAPKs and PLC pathways in the ICCs.

## 1. Introduction

Interstitial cells of Cajal (ICCs) are the pacemaking cells in the gastrointestinal (GI) muscles that generate the rhythmic oscillations in the membrane potential known as slow waves [1–3]. Slow waves propagate within ICC networks, are conducted into smooth muscle cells via gap junctions, and initiate phasic contractions by activating Ca^2+^ entry through L-type Ca^2+^ channels. The pacemaker activity in the murine small intestine is due mainly to periodic activation of nonselective cation channels (NSCC) [4, 5] or Cl^−^ channels [6, 7]. ICCs also mediate or transduce inputs from the enteric nervous system. Therefore, in GI motility research, ICCs are the major tools to study. In these days, many new drugs are developing in the area of GI motility.

From ancient to modern history, traditional plant-based medicines have played an important role in health care. In spite of the great advances of modem scientific medicine, traditional medicine is still the primary form of healing methods readily available to the majority of the people in many countries. In fact many of today's popular drugs have their origins in traditional medicines [8].

So-Cheong-Ryong-Tang (SCRT), also called Xiao-Qing-LongTtang or Sho-Seiru-To, contains eight species of medicinal plants and has been a herbal medicine used to treat diseases such as allergic rhinitis and asthma for hundreds of years in Asian countries [9]. However, there were not many attempts to investigate the efficacy of SCRT in digestive systems.

Of the pathways related to intestinal motility, serotonin (5-hydroxytryptamine, 5-HT; a major neuromodulator) is known to play a critical role in the GI tract. Generally, 5-HT acts as a neurotransmitter in the central nervous system, but most (95%) 5-HT is found in the GI tract [10]. Furthermore, although 5-HT is known to interact with seven different 5-HT receptor subtypes, only three of these are found in the ICCs in the murine small intestine [11]. 5-HT can modulate pacemaker activity through 5-HT3, 4, and 7 receptors. In previous study, we suggested that poncirus trifoliate modulates pacemaker potentials through 5-HT_3_ and 5-HT_4_ receptor-mediated pathways via external Na^+^ and Ca^2+^ influx [6]. However, the effects of SCRT and the action mechanism involved in the GI tract are not investigated.

Therefore, we undertook to investigate the effects of the SCRT on the pacemaker potentials of cultured ICCs derived from murine small intestine and to identify the receptors involved.

## 2. Materials and Methods

### 2.1. Preparation of Cells and Cell Cultures

Balb/c mice (3–7 days old) of either sex were anesthetized with ether and killed by cervical dislocation. The small intestines from 1 cm below the pyloric ring to the cecum were removed and opened along the mesenteric border. Luminal contents were removed by washing with Krebs-Ringer bicarbonate solution. The tissues were pinned to the base of a Sylgard dish and the mucosa removed by sharp dissection. Small tissue strips of the intestine muscle (consisting of both circular and longitudinal muscles) were equilibrated in Ca^2+^-free Hanks solution (containing in mmol/L: KCl 5.36, NaCl 125, NaOH 0.34, Na_2_HCO_3_ 0.44, glucose 10, sucrose 2.9, and HEPES 11) for 30 min. Then, the cells were dispersed using an enzyme solution containing collagenase (Worthington Biochemical Co., Lakewood, NJ, USA) 1.3 mg/mL, bovine serum albumin (Sigma Chemical Co., St. Louis, MO, USA) 2 mg/mL, trypsin inhibitor (Sigma) 2 mg/mL and ATP 0.27 mg/mL. Cells were plated onto sterile glass coverslips coated with murine collagen (2.5 *μ*g/mL, Falcon/BD, Franklin Lakes, NJ, USA) in a 35-mm culture dish and then cultured at 37°C in a 95% O_2_, 50 mL/L CO_2_ incubator in a smooth muscle growth medium (Clonetics Corp., San Diego, CA, USA) supplemented with 2% antibiotics/antimycotics (Gibco, Grand Island, NY, USA) and murine stem cell factor (SCF, 5 ng/mL, Sigma). ICCs were identified immunologically with anti-c-kit antibody (phycoerythrin-conjugated rat anti-mouse c-kit monoclonal antibody; eBioscience, San Diego, CA, USA) at a dilution of 1 : 50 for 20 min [7]. ICCs were morphologically distinct from other cell types in the culture and thus it was possible to identify the cells by phase contrast microscopy once they had been verified with anti c-kit antibody.

### 2.2. Patch-Clamp Experiments

The whole-cell patch-clamp configuration was used to record membrane potentials (current clamp) from cultured ICCs. An axopatch ID (Axon Instruments, Foster, CA, USA) was used to amplify membrane currents and potentials. The command pulse was applied using an IBM-compatible personal computer and pClamp software (version 6.1; Axon Instruments). Data obtained were filtered at 5 kHz and displayed on an oscilloscope, a computer monitor, and using a pen recorder (Gould 2200, Gould, Valley View, OH, USA). Results were analyzed using pClamp and Origin (version 6.0) software. All experiments were performed at 30–32°C.

### 2.3. Fura-2 Loading and Measurement of Intracellular Free Calcium Ion Concentration [Ca^2+^]_**i**_

Cultured ICC clusters were loaded with the acetoxymethyl ester form of fura-2 (5 *μ*mol/L; diluted from 1 mmol/L stock in dimethyl sulfoxide (DMSO)) in normal medium for 20 minutes at 37°C. The recording of [Ca^2+^]_i_ was performed with a microfluorometric system consisting of an inverted fluorescence microscope (Diaphot 300; Nikon, Japan) with a dry-type fluorescence objective lens (40X; numerical aperture 0.85), a photomultiplier tube (type R 1527; Hamamatsu, Japan), and a PTI-Deltascan illuminator (Photon Technology International Inc.). Cells were superfused at a flow rate of 1.5 mL/min. Light was provided by a 75-W xenon lamp (Ushino, Japan). To control excitation frequency, a chopper wheel alternated the light path to monochromators (340 and 380 nm) with a frequency of 5 or 10 Hz. A short-pass dichroic mirror passed emission light of <570 nm onto the photomultiplier tube, and intensity was measured at 510 nm. A mechanical image mask was placed in the emission path to limit measurement to a single cell. Both data acquisition and control of light application were performed with computer software (Felix version 1.1; Photon Technology International Inc., Brunswick, NJ). Because of uncertainties in calibrating the fura-2 signals in intact cells, no attempt was made to calibrate [Ca^2+^]_i_; instead, all results are reported as changes in the 340 nm/380 nm signal ratio.

### 2.4. Solutions and Drugs

The physiological salt solution used to bathe cells (Na^+^-Tyrode) contained (mmol/L): KCl 5, NaCl 135, CaCl_2_ 2, glucose 10, MgCl_2_ 1.2, and HEPES 10, adjusted to pH 7.4 with NaOH. The pipette solution contained (mmol/L): KCl 140, MgCl_2_ 5, K_2_ATP 2.7, NaGTP 0.1, creatine phosphate disodium 2.5, HEPES 5, and EGTA 0.1, adjusted to pH 7.2 with KOH.

SCRT was purchased from I-WORLD Pharmaceuticals (South Korea). SCRT is composed of Rhizoma Pinelliae, Herba Ephedrae, Radix Paeoniae, Fructus Schisandrae, Herba Asari, Rhizoma Zingiberis, Ramulus Cinnamomi, and Radix Glycyrrhizae (Table 1). The dosage for adult is 10–15 g (crude materials) per day. More information about SCRT can be found in I-WORLD Pharmaceuticals Homepage (http://i-pharm.koreasme.com/). The SCRT was dissolved with distilled water at the concentration of 0.5 g (crude drug)/mL and stored in refrigerator. All other drugs were obtained from Sigma (Sigma Chemical Co., USA). Drugs were dissolved in distilled water and added to bath solution to make the desired concentrations, just prior to use. Addition of these chemicals to bath solution did not alter the pH of the solution. U-73122, U-73343, Y25130, RS39604, and SB269970 were dissolved in DMSO for 50 mmol/L stock solution and added to the bathing solution at the day of the experiment. The final concentration of DMSO in the bath solution was always <0.1%, and we confirmed that this concentration of DMSO did not affect the results that were recorded.

### 2.5. Statistics

All data are expressed as mean ± S.E. Student's *t*-test for unpaired data was used to compare control and experimental groups. The *P* value of less than 0.05 was considered statistically significant.

## 3. Results

### 3.1. Effect of SCRT on Pacemaker Potentials in Cultured ICCs

**Figure 1:**
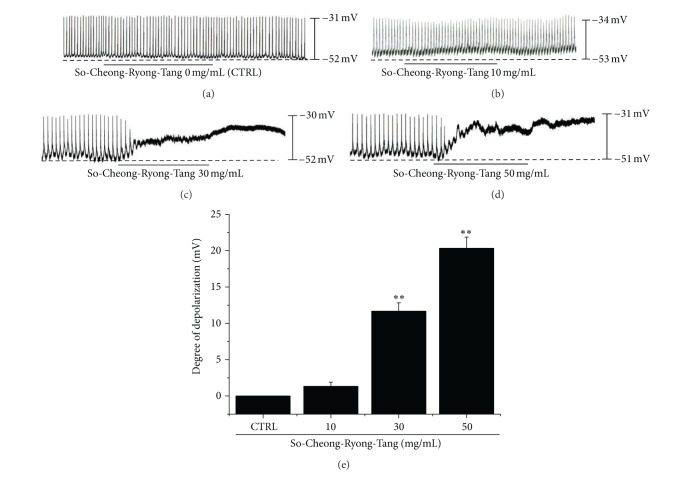
Effects of SCRT on pacemaker potentials in cultured ICCs from murine small intestine. (a)–(d) show the pacemaker potentials of ICCs exposed to SCRT (0–50 mg/mL) in current clamping mode (*I* = 0). Responses to SCRT are summarized in (e). Bars represent mean values ± SEs. **P < 0.01. Significantly different from untreated controls. CTRL: Control.

To understand the relationship between SCRT and pacemaking activity in ICCs, we examined the effects of SCRT on pacemaker potentials. Recording from cultured ICC under current clamp mode (*I* = 0) showed spontaneous pacemaker potentials. The resting membrane potential was −52.1 ± 2.1 mV, and the amplitude was 20 ± 4 mV. In the presence of SCRT (10–50 mg/mL), the membrane potentials were depolarized to 1.3 ± 0.6 mV at 10 mg/mL, 11.6 ± 1.2 mV at 30 mg/mL, and 20.3 ± 1.5 mV at 50 mg/mL (Figures 1(a)–1(e)). The summarized values and bar graph of the SCRT effects on pacemaker potentials are indicated in Figure 1(e) (*n* = 4). Taken together, these results show that SCRT have membrane depolarization effects on ICC.

### 3.2. Identification of SCRT Receptor Subtypes in Cultured ICCs

**Figure 2:**
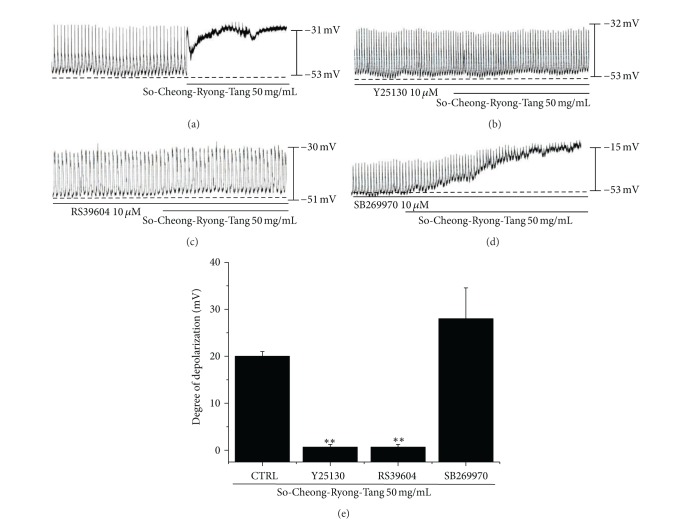
Effects of 5-HT receptor subtype antagonists on SCRT-induced pacemaker potential responses in cultured ICCs. (a) SCRT (50 mg/mL) induced membrane depolarizations on ICCs. (b) Pacemaker potentials of ICCs exposed to SCRT (50 mg/mL) in the presence of 5-HT_3_ receptor antagonist (Y25130; 10 *μ*M). (c) Pacemaker potentials of ICCs exposed to SCRT in the presence of 5-HT_4_ receptor antagonist (RS39604; 10 *μ*M). (d) Pacemaker potentials of ICCs exposed to SCRT in the presence of 5-HT_7_ receptor antagonist SB269970 (10 *μ*M). Responses to SCRT in presence of different receptor antagonists are summarized in (e). Bars represent mean values ± SEs. **P < 0.01. Significantly different from untreated controls. CTRL: Control.

To investigate the relationship between SCRT and its receptors, we studied about the 5-HT receptors because 5-HT receptors are known to mediate the motility of GI tract and is of particular interest due to its strong association with potent prokinetic activity, especially the 5-HT receptor subtype 4 (5-HT_4_R) [6, 12]. In the GI tract, the stimulation of 5-HT_4_R in the enteric nervous system results in the release of acetylcholine, which leads to the excitation of smooth muscles in the myenteric plexus, and thus, 5-HT_4_R is regarded a prokinetic [12]. Therefore, we investigated whether the prokinetic action of SCRT involves 5-HT receptors. Previous studies have shown that 5-HT interacts with seven different 5-HT receptor subtypes, but in another study only three (5-HT_3_R, 5-HT_4_R, and 5-HT_7_R) were found in the ICCs of the murine small intestine [6, 11, 13]. To identify the receptor subtypes of 5-HT involved in the effects of SCRT, ICCs were pretreated with various 5-HT receptor antagonists and then treated with SCRT. Y25130 (a 5-HT_3_ receptor antagonist), RS39604 (a 5-HT_4_ receptor antagonist), and SB269970 (a 5-HT_7_ receptor antagonist) were all pretreated at a concentration of 10 *μ*M for 5 min, and SCRT was added. After pretreatment with Y25130, membrane depolarization by SCRT was found to be blocked (Figure 2(b)); membrane depolarization produced in the presence of Y25130 by MPF was 1.0 ± 0.2 mV (*n* = 5; Figure 2(e)). RS39604 also blocked SCRT-induced membrane depolarization, and the membrane depolarization produced in the presence of RS39604 by SCRT was 1.2 ± 0.3 mV (*n* = 5; Figures 2(c) and 2(e)). However, pretreatment with SB269970 did not block the effect of SCRT (*n* = 5; Figures 2(d) and 2(e)). These results show that SCRT has an effect on ICCs through 5-HT_3_R and 5-HT_4_R.

### 3.3. The Involvement of G Protein on SCRT-Induced Depolarizations in Pacemaker Potentials in Cultured ICCs

**Figure 3:**
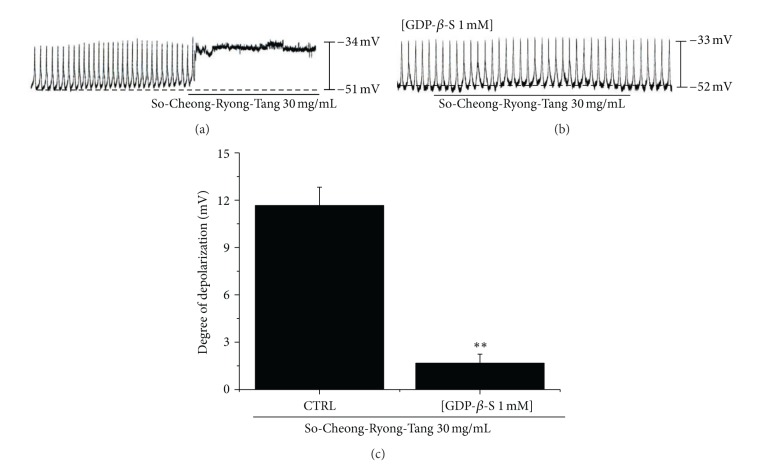
Effects of GDP-*β*-S in the pipette on SCRT-induced pacemaker potentials in cultured ICCs of murine small intestine. (a) SCRT (30 mg/mL) induced membrane depolarizations on ICCs. (b) Pacemaker potentials of ICCs exposed to SCRT (30 mg/mL) in the presence of GDP-*β*-S (1 mM) in the pipette. Under these conditions, SCRT (30 mg/mL) caused membrane depolarization. (c) Responses to SCRT in the presence of GDP-*β*-S in the pipette are summarized in (b). Bars represent mean values ± SEs. **P < 0.01. Significantly different from untreated controls. CTRL: Control.

The effects of GDP-*β*-S (a nonhydrolysable guanosine 5′-diphosphate analogue that permanently inactivates G-protein binding proteins [14]) were examined to determine whether G-proteins are involved in the effects of SCRT on cultured ICCs. SCRT (30 mg/mL) induced membrane depolarizations on ICCs (Figure 3(a)). However, when GDP-*β*-S (1 mM) was in the pipette solution, SCRT (30 mg/mL) did not induce the membrane depolarizations (Figure 3(b)). The membrane depolarizations induced by SCRT were significantly affected by the presence of GDP-*β*-S (1 mM) in the pipette solution (*n* = 4, Figure 3(c)). These results show that G-protein is involved in SCRT-induced membrane depolarizations on ICCs.

### 3.4. Response of the Intracellular Ca^2+^ ([Ca^2+^]_**i**_) to SCRT

**Figure 4:**
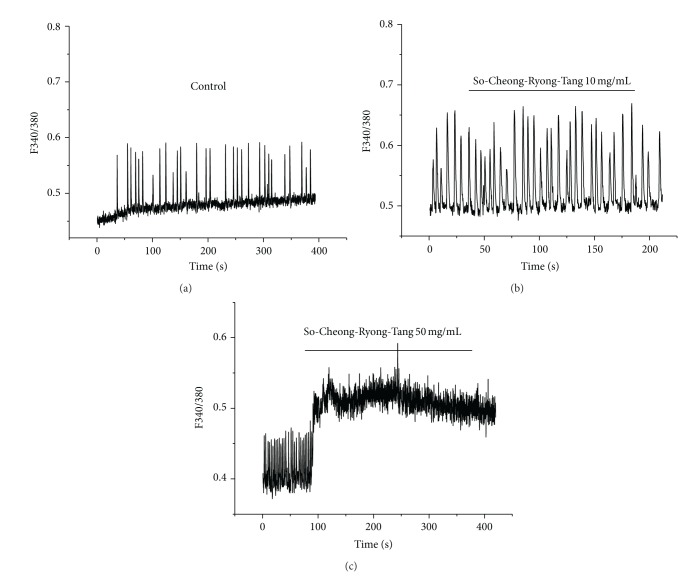
Responses of intracellular Ca^2+^ to SCRT in cultured ICCs. ICCs were suspended in 2 mM Ca^2+^-containing normal solution. Increase in [Ca^2+^]_i_ was plotted against SCRT concentrations. (a) In normal conditions, [Ca^2+^]_i_ oscillations was induced. (b) SCRT 10 mg/mL had no response, but SCRT 50 mg/mL caused a sustained increase in [Ca^2+^]_i_ (c).

To investigate the effects of SCRT on [Ca^2+^]_i_ oscillations, we measured spontaneous [Ca^2+^]_i_ oscillations in ICCs clusters because several authors have suggested that [Ca^2+^]_i_ oscillations in ICCs are primary responsible for GI pacemaker activity. Spontaneous [Ca^2+^]_i_ oscillations were observed in ICCs clusters loaded with 5 *μ*M fluo-2.  Figure 4 show the changes in the 340 nm/380 nm signal ratio. In normal conditions, spontaneous [Ca^2+^]_i_ oscillations were induced (Figure 4(a)). In the presence of SCRT (10 or 50 mg/mL), [Ca^2+^]_i_ in ICCs was increased (Figures 4(b) and 4(c)). These results show that SCRT increase the [Ca^2+^]_i_ in ICCs.

### 3.5. Effects of Phospholipase C Inhibitor on SCRT-Induced Depolarizations in Pacemaker Potenials in Cultured ICCs

**Figure 5:**
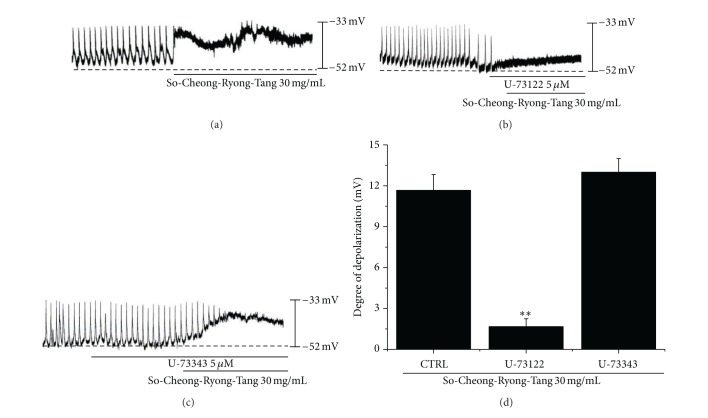
Effects of SCRT on phospholipase C inhibitors in cultured ICCs. (a) SCRT (30 mg/mL) induced membrane depolarizations on ICCs. (b) U-73122 (5 *μ*M), a phospholipase C inhibitor, abolished the generation of pacemaker potentials. U-73122 blocked the SCRT-induced (30 mg/mL) membrane depolarizations. (c) The application of U-73343 (5 *μ*M) did not show any influence on the generation of pacemaker currents. Also, U-73343 did not block the SCRT-induced (30 mg/mL) membrane depolarizations. (d) The responses to SCRT in phospholipase C inhibitors are summarized in (d). Bars represent mean values ± SEs. **P < 0.01. Significantly different from untreated controls. CTRL: Control.

Since the membrane depolarizations by SCRT was related to intracellular Ca^2+^ mobilization, we examined whether the effects on pacemaker potentials require phospholipase C (PLC) activation. To test this possibility, SCRT-induced membrane depolarizations were measured in the absence and presence of U-73122, an active PLC inhibitor [15]. SCRT (30 mg/mL) induced membrane depolarizations on ICCs (Figure 5(a)). The pacemaker membrane depolarizations were completely abolished by application of U-73122 (5 *μ*M), and under these conditions, SCRT-induced membrane depolarizations were not produced (*n* = 4; Figure 5(b)). In the presence of U-73122, the membrane depolarizations produced by SCRT were 1.7 ± 0.6 mV. The value of membrane depolarizations by SCRT was significantly different when compared with SCRT in the absence of U-73122 (*n* = 4, Figure 5(d)). The treatment of U-73343 (5 *μ*M), an inactive analog of U-73122, had no influence on the pacemaker potentials, and under these conditions, SCRT-induced membrane depolarizations were not suppressed by U-73343 (Figure 5(c)). These results show that PLC pathway is involved in SCRT-induced membrane depolarizations on ICCs.

### 3.6. Involvements of Mitogen-Activated Protein Kinases (MAPKs) in SCRT-Induced Depolarizations in Pacemaker Potentials in Cultured ICCs

**Figure 6:**
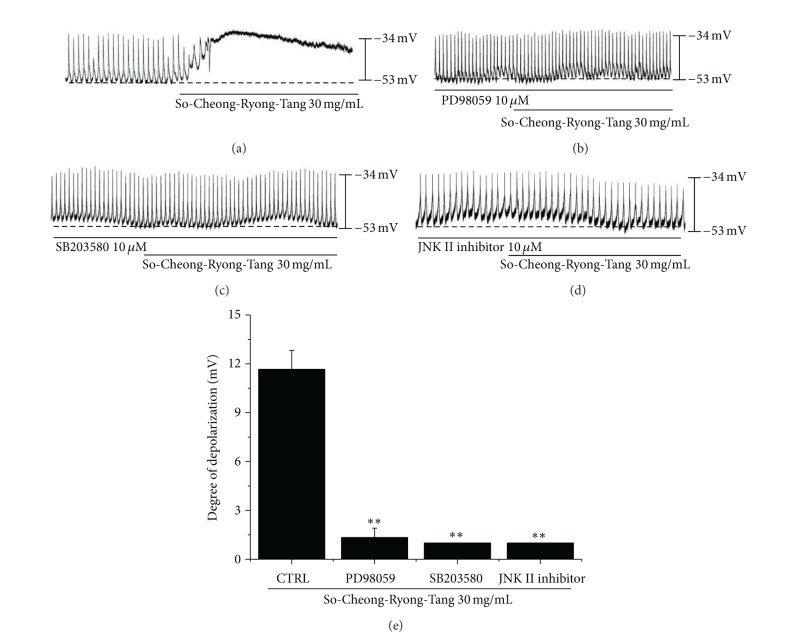
Effects of various MAPK inhibitors on SCRT-induced pacemaker potentials responses in cultured ICCs. (a) SCRT (30 mg/mL) induced membrane depolarizations on ICCs. (b) Pacemaker potentials of cultured ICCs exposed to SCRT (30 mg/mL) in the presence of 10 *μ*M of PD98059 (a p42/44 MAPK inhibitor). (c) Pacemaker potentials of cultured ICCs exposed to SCRT in the presence of 10 *μ*M of SB203580 (a p38 MAPK inhibitor). (d) Pacemaker potentials of an ICC exposed to SCRT in the presence of 10 *μ*M of JNK II inhibitor. Responses to SCRT in the presence of different MAPK inhibitors are summarized in (e). Bars represent mean values ± SEs. **P < 0.01. Significantly different from untreated controls. CTRL: Control.

Approximately 90% of endogenous 5-hydroxytryptamine (5-HT) in the body exists in the digestive tract and 5-HT is believed to be involved in the regulation of gastrointestinal motility. Also, it has been reported that 5-HT activates MAPKs in many cell types, and thus we examined whether or not MAPKs are involved in the effects induced by SCRT by using PD98059 (a p42/44 MAPK inhibitor), SB203580 (a p38 MAPK inhibitor), or c-jun NH2-terminal kinase (JNK) II inhibitor. SCRT (30 mg/mL) induced membrane depolarizations on ICCs (Figure 6(a)). In the presence of PD98059 (10 *μ*M), SCRT did not produce membrane depolarizations (*n* = 4; Figures 6(b) and 6(e)), which indicated that p42/44 plays a role in SCRT-induced membrane depolarization. In addition, SB203580 and JNK II inhibitor blocked the depolarizations by SCRT in pacemaker potentials (*n* = 4; Figures 6(c), 6(d) and 6(e)). These results show that the regulation of mitogen-activated protein kinases is involved in SCRT induced membrane depolarizations on ICCs.

## 4. Discussion

In recent years, there has been much work done in the area of traditional medicinal plants in Korea and many good results or promising leads have been achieved [8]. In GI motility area, ICCs are good research tools to study GI motility and therefore, many labs use ICCs.

In this study, we used ICCs and are studying to develop a new GI motility drug. Until now, SCRT was not used to treat GI motility diseases. SCRT has been widely used to treat respiratory disease such as cough and asthma in oriental countries [16]. In this study, we found the potentials of SCRT to treat in GI motility. Because of the central role of ICCs in GI motility, loss of these cells would be extremely detrimental. Research into the biology of ICCs provides exciting new opportunities to understand the etiology of diseases that have long eluded comprehension. Discovering the molecules involved in the generation of pacemaker activity in ICCs may lead to dramatic new therapies for chronic GI diseases that result in lifelong suffering. Consequently, ICCs are involved not only in physiological GI motility but also in many bowel disorders, including inflammatory bowel disease, chronic idiopathic intestinal pseudo-obstruction, intestinal obstruction with hypertrophy, achalasia, Hirschsprung disease, juvenile pyloric stenosis, juvenile intestinal obstruction, and anorectal malformation [17].

5-HT plays a critical role in coordinating GI motility [18–21]. In response to intestinal stretching stimulation, 5-HT promotes peristalsis via the activation of intrinsic primary afferent neurons located in the submucous plexus [12, 22, 23]. Of the 5-HT receptors believed to influence GI motility, 5-HT_3_ and 5-HT_4_ receptors play an important role in the regulation of GI motility [12, 24–26]. Also, in guinea pig colon, 5-HT receptor was investigated [20]. Furthermore, these receptors mediate peristaltic reflex and existed in nervous systems in the guinea pig distal colon [27]. Liu et al. [13] suggested that 5-HT augments ICC pacemaker activity via 5-HT3 receptors, whereas Shahi et al. [11] suggested that 5-HT can modulate pacemaker activity via 5-HT3, 4, and 7 receptors. On the other hand, Wouters et al. [28] suggested that 5-HT2B receptors regulate the proliferation of ICCs in mouse jejunum. In Korea, poncirus trifoliate (PT) is widely used as a remedy for GI, allergic, and inflammatory diseases [29, 30]. In recent study, Kim et al. [6] suggested that PT exerts its prokinetic activity through a 5-HT_3_ and 5-HT_4_ receptor-mediated pathway in ICCs in mouse small intestine. In the present study, Y25130 (a 5-HT_3_ receptor antagonist) and RS39604 (a 5-HT_4_ receptor antagonist) blocked SCRT-induced membrane depolarizations, whereas SB269970 (a 5-HT_7_ receptor antagonist) did not. Thus, it appears that SCRT modulates pacemaker potentials through 5-HT_3_ and 5-HT_4_ receptors-mediated pathways in the ICCs of mouse small intestine (Figure 2).

In this study, ICCs generated pacemaker potentials in mouse small intestine. SCRT produced membrane depolarization in current clamp mode. Y25130 (a 5-HT_3_ receptor antagonist) and RS39604 (a 5-HT_4_ receptor antagonist) blocked MPF-induced membrane depolarizations, whereas SB269970 (a 5-HT_7_ receptor antagonist) did not. When GDP-*β*-S (1 mM) was in the pipette solution, SCRT did not induce the membrane depolarizations. Also, SCRT increased intracellular Ca^2+^ concentrations. To examined whether or not MAPKs are involved in the effects induced by SCRT, we used PD98059 (a p42/44 MAPK inhibitor), SB203580 (a p38 MAPK inhibitor), or c-jun NH2-terminal kinase (JNK) II inhibitor. In the presence of PD98059 (10 *μ*M), SCRT did not produce membrane depolarizations. In addition, SB203580 and JNK II inhibitor blocked the depolarizations by SCRT in pacemaker potentials. Furthermore, the membrane depolarizations by SCRT were inhibited not by U-73122, an active phospholipase C (PLC) inhibitors but by U-73343, an inactive PLC inhibitors. These results suggest that SCRT might affect GI motility by the modulation of pacemaker activity through MAPKs and PLC pathways in the ICCs.

**Figure 7:**
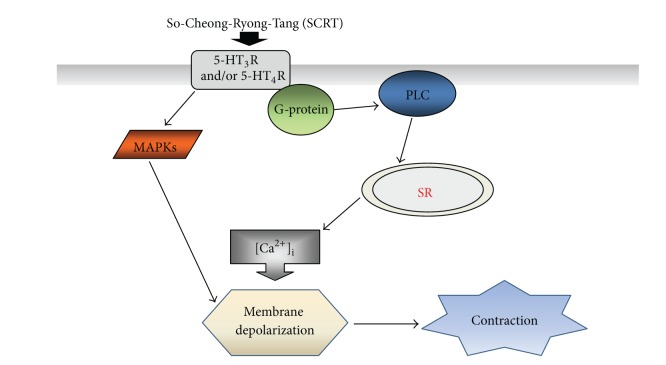
Hypothetical schematic signaling pathway of SCRT-induced membrane depolarizations on ICCs. SCRT-induced membrane depolarization seems to be mediated by 5-HT_3_ and/or 5-HT_4_ receptor, coupled to G protein, resulting in the activation of PLC pathway, which subsequently increases the intracellular Ca^2+^ level from SR. The data also suggest that MAPKs are involved in the SCRT-induced membrane depolarizations.

We think that SCRT activates PLC pathway (Figure 5) and increases intracellular Ca^2+^ levels (Figure 4). Intracellular Ca^2+^ increases induce the membrane depolarizations on ICCs and then make ICCs contractions (Figure 7). However, the exact mechanisms of membrane depolarizations on ICCs should be investigated in future.

The signaling pathways of MAPKs play important roles in the mediation of cellular responses, including visceral smooth muscle contraction. Three principal MAPKs are expressed in various tissues (p42/44, JNK, and p38 MAPK) [31]. Furthermore, it has been demonstrated 5-HT_7_ receptors activate MAP kinase in hippocampal neurons [32] and that 5-HT induces cyclooxygenase (COX)-2 by activating MAPK in vascular smooth muscle cells [33], which show that 5-HT can regulate the MAPK system. In the present study, we do not know whether this SCRT has a role of 5-HT or not. However, in this study, an inhibitor of p42/44 and p38 and a JNK II inhibitor inhibited the effect of SCRT, which suggests that p38, p42/44, and JNK MAPK are involved in the modulation of pacemaker potentials by SCRT.

Taken together, our data suggest that the SCRT have an ability to regulate the pacemaker potentials in ICCs and considering the effects of this drug on the ICC, further research, including finding active compound(s) and examining their action mechanisms, is clearly needed.

## Figures and Tables

**Table 1:** Amount and Composition of SCRT.

Herb	Scientific name	Amount (g)
Ban Ha	Rhizoma Pinelliae	2 g
Ma Hwang	Herba Ephedrae	1 g
Jak Yak	Radix Paeoniae	1 g
Omija	Fructus Schisandrae	1 g
Sae Shin	Herba Asari	1 g
Gun Kang	Rhizoma Zingiberis	1 g
Kae Ji	Ramulus Cinnamomi	1 g
Kam Cho	Radix Glycyrrhizae	1 g

Total amount		9 g
